# Survival in Adult Patients Undergoing Heart Transplantation 1995–2024: A Report of the RETRAC Registry

**DOI:** 10.5334/gh.1520

**Published:** 2026-02-03

**Authors:** Juan David López-Ponce-de-León, Juan Andrés Muñoz-Ordoñez, Alejandro Toro-Pedroza, Juan Pablo Arango-Ibanez, Valeria Azcarate-Rodriguez, María Camila Naranjo-Ramírez, Hoover León-Giraldo, Jessica Largo, Diana Carrillo-Gomez, Andrea Alejandra Arteaga-Tobar, Manuela Escalante-Forero, Pastor Olaya, Noel Florez, Nancy Olaya, Edilma Lucy Rivera-Muñoz, Mario Miguel Barbosa-Rengifo, Jose Nativi-Nicolau, Juan Esteban Gómez-Mesa

**Affiliations:** 1 Servicio de Cardiología, Fundación Valle del Lili, Cali, Valle del Cauca, Colombia; 2 Facultad de Ciencias de la Salud, Universidad Icesi, Cali, Valle del Cauca, Colombia; 3 Centro de Investigaciones Clínicas, Fundación Valle del Lili, Cali, Valle del Cauca, Colombia; 4 Division of Advanced Heart Failure and Transplantation, Department of Transplantation, Mayo Clinic, Jacksonville, FL, USA

**Keywords:** heart transplantation, heart failure, survival analysis, Colombia, chronic kidney disease, RETRAC registry

## Abstract

**Background:**

Heart transplantation (HT) remains the definitive treatment for advanced heart failure that is refractory to both medical and invasive therapies. Although global registries offer extensive data on survival outcomes, there is a relative paucity of information regarding HT outcomes in Latin America (LATAM), particularly in Colombia.

**Methods:**

This study analyzed adult patients who underwent HT between 1995 and 2024, using data obtained from an institutional HT registry (RETRAC) in Cali, Colombia. Survival outcomes were evaluated using Kaplan–Meier curves and Cox proportional hazards models.

**Results:**

We included 260 patients who underwent HT in this 29-year cohort from a LATAM country. The median age at transplantation was 51 years, and 77.7% were male. The primary etiologies were idiopathic/dilated cardiomyopathy (IDC) (41.3%), ischemic cardiomyopathy (IC) (27.0%), and valvular heart disease (VHC) (9.7%). The most prevalent comorbidities were hypertension (HTN) (48.3%), diabetes mellitus (DM) (18.9%), and chronic kidney disease (CKD) (13.1%). The overall median survival following HT was 7.4 years. One-year survival was 74.6% (n = 194), five-year survival was 56.9% (n = 147), and ten-year survival was 46.9% (n = 122). Survival differed significantly by age and sex, with patients aged <40 years demonstrating the highest median survival (8.4 years) and those aged ≥60 years the lowest (2.2 years) (p = 0.038). The 40- to 49-year age group exhibited the most pronounced reduction in survival; however, this effect was attenuated after adjustment. Among patients under 40 years, females had significantly higher mortality compared to males (p = 0.0078), with younger males exhibiting better survival. Additionally, patients transplanted between 2016 and 2020 had higher survival rates. CKD was identified as a significant independent risk factor for increased mortality (hazard ratio (HR) = 1.79; 95% CI: 1.15–2.79; p = 0.01).

**Conclusions:**

HT patients in Colombia exhibit demographic and clinical profiles comparable to global cohorts; however, they demonstrate lower survival rates and poorer clinical outcomes compared to international registries, such as the International Society for Heart and Lung Transplantation registry. Nonetheless, clinical outcomes are more favorable than those reported in other studies from the LATAM region. CKD emerged as a significant independent predictor of mortality. These findings highlight the need for region-specific strategies aimed at improving HT outcomes in LATAM.

## Visual Abstract

**Figure:**
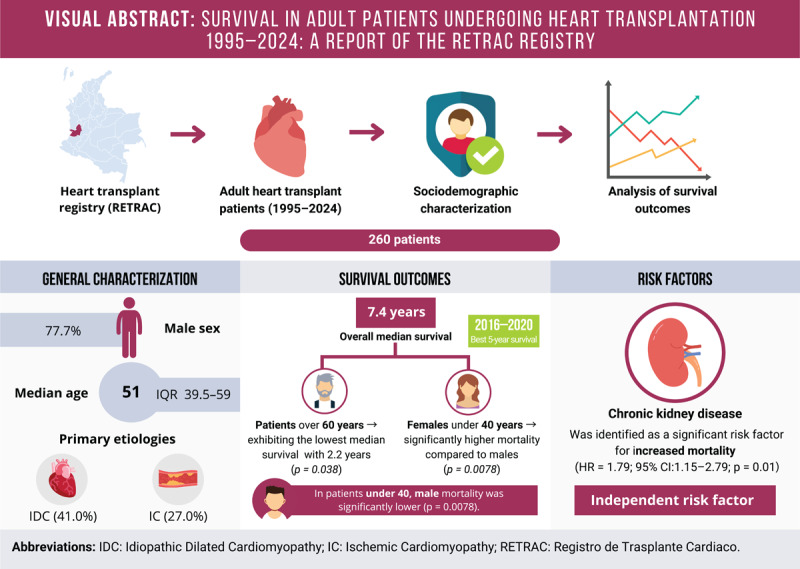


## Introduction

Heart failure (HF) is a major global health concern, affecting approximately 64.3 million individuals worldwide, and has been described as a global pandemic (1). For patients with advanced HF who are unresponsive to optimal medical therapy, device support, and surgical interventions, heart transplantation (HT) remains a treatment option (2,3,4). According to the International Society for Heart and Lung Transplantation (ISHLT) Thoracic Organ Transplant Registry, the median survival following HT is 11.3 years, increasing to 13.2 years among patients aged 18–39 years and decreasing progressively with advancing age (5). Despite significant progress in cardiac critical care, immunosuppressive therapy, and chronic HF management, further research is warranted to identify factors influencing long-term survival and outcomes in HT recipients, particularly in Latin American (LATAM) populations and specifically in Colombia, where available data remain limited.

Between 1992 and 1997, the ISHLT registry reported a total of 97,140 HT procedures performed globally, with the majority conducted in North America (51.8%) and Europe (41.1%). The leading etiologies were non-ischemic cardiomyopathy (48.1%) and ischemic cardiomyopathy (IC) (40.6%) (5). Data on HT in LATAM remain limited, with most available information originating from Brazil, where 3,415 HT procedures were recorded between 2011 and 2021, including 332 in 2021 (6). A study from Uruguay reported a significant increase in HT rates, rising from an average of 4.3 per million population during 1989–1998 to 29.38 per million during 1999–2008 (7). In contrast, in Colombia, a cohort of 579 HT patients reported post-transplant survival rates at 1, 5, and 10 years of 71.3%, 50.7%, and 36.5%, respectively (8). However, much of the existing data lack detailed survival analysis for HT patients. To address this gap, we previously analyzed the Registro de Trasplante Cardíaco (RETRAC), reporting a first-year mortality rate of 24% (9). Based on this, we conducted a study to evaluate survival trends over time and improve understanding of HT outcomes in Colombia. These insights aim to guide clinical strategies tailored to the regional patient population.

## Methods

### Study design

This study analyzes a cohort of HT patients enrolled in the RETRAC registry, an observational, ambispective, single-center study designed to characterize the demographic and clinical features of HT patients followed at Fundación Valle del Lili (FVL), a high-complexity institution in Cali, Colombia. The Centro de Investigaciones Clínicas (CIC) of this institution coordinated and supervised the study.

### Participants

The RETRAC registry includes patients of all ages who have undergone HT and have been managed and followed at FVL. Notably, not all patients included in the registry underwent HT at our institution; some were enrolled in RETRAC approximately 1–3 months after transplantation performed elsewhere. The registry comprises data from outpatient records, emergency department visits, and hospitalization records related to HT and post-transplant outcomes. For patients who underwent re-HT, only the first transplant episode was considered the index procedure and the starting point for follow-up. In cases of multi-organ transplantation, the procedure was analyzed as an HT for the purposes of this study. Thus, analyses were restricted to first events, without accounting for recurrent outcomes.

Patient recruitment began in 1995 and remains ongoing. For this study, we included all adults aged 18 years or older and recruited between 1995 and 2024, regardless of the transplant date. Data collection was conducted retrospectively until 2022, after which it has been conducted prospectively. Patients younger than 18 years, those lost to follow-up, or those with incomplete data were excluded.

### Data collection

Data were collected from institutional electronic medical records. Sociodemographic characteristics, comorbidities, and medical history were recorded at the time of the patient’s initial assessment at the institution. Donor information and peri-procedural data specific to the transplant episode were also extracted from institutional records. The suspected etiology of HF, or the underlying cause leading to HT, was determined based on the patient’s medical history and comprehensive diagnostic evaluations. Furthermore, we verified the current vital status (alive or deceased) of the patients using the ‘*Administradora de los Recursos del Sistema General de Seguridad Social en Salud*’ database, with a cutoff date of February 2025. The date of death or last known alive date from this source was used to define censoring times in the survival analyses.

All data were securely stored using the Research Electronic Data Capture (REDCap) platform.

### Variable definitions

Comorbidities were ascertained from the medical records and recorded at the time of the comprehensive pre-HT evaluation. Hypertension (HTN) was defined as the use of antihypertensive medication prior to HT. Diabetes mellitus (DM) was defined as the use of antidiabetic medication prior to HT. Chronic kidney disease (CKD) was defined as a documented diagnosis of CKD, an estimated glomerular filtration rate <60 mL/min/1.73 m^2^, or the requirement of renal replacement therapy prior to transplantation.

Other comorbidities, including chronic obstructive pulmonary disease, deep venous thrombosis, pulmonary embolism, occlusive arterial disease, stroke, transient ischemic attack, and neoplasia, were identified based on documented clinical history and physician assessment during the pre-HT evaluation, supported by a comprehensive diagnostic workup as part of standard transplant candidate assessment. The etiology of HF was determined by the treating cardiologist based on clinical history, imaging, hemodynamic assessments, and biopsy findings, when available or deemed necessary according to clinical judgment.

### Statistical analysis

The Kolmogorov–Smirnov test was used to assess the normality of continuous variables, which were presented as medians with interquartile ranges or as means with standard deviations. Categorical variables were reported as absolute numbers and percentages. Kaplan–Meier survival curves were generated to illustrate post-HT survival, and the log-rank test was used to compare survival curves. Confounder-adjusted survival curves were estimated using direct standardization (G-computation) via the adjusted curves R package (10,11). This method fits a Cox proportional hazards model with confounding variables, predicts survival probabilities for each patient under different exposure categories, and averages these predictions to obtain marginal survival curves. The model included DM and CKD as confounders, allowing age-stratified survival curves adjusted for differential comorbidity distribution across age groups.

A Cox proportional hazards model was constructed to evaluate mortality risk factors in our HT population. We developed three models. First, we conducted a univariate analysis. Then, we used a backward elimination process considering variables with a p-value of ≤0.25 in the univariate analysis to create models 1 and 2:

- Saturated model (Model 1), which included variables such as age group (missing: 0.0%), history of DM (missing: 0.4%), history of vasoactive support (missing: 1.5%), sex (missing: 0.0%), history of CKD (missing: 1.9%), ischemic time (missing: 2.3%), and history of underlying heart disease (missing: 0.0%).
- Parsimonious model (Model 2), which included age group (missing: 0.0%), DM (missing: 0.4%), and CKD (missing: 1.9%).

Additionally, both global and individual Schoenfeld residual tests were conducted to assess the proportional hazards assumption and potential time dependence within the model. A p-value threshold of <0.05 was used for this analysis. The parsimonious model included three variables, with an adequate events-per-variable ratio of 53. Further details regarding the events per variable are provided in Supplementary 1. Variables with ≥20% missing data were excluded. Missing data were addressed using complete case analysis; no imputation procedures were applied. Additional information on the extent of missing data across registry variables is presented in Supplementary 2. Multicollinearity among predictor variables was assessed using the generalized variance inflation factor (GVIF); no variables exhibited a GVIF >1.2 (Supplementary 3). Non-linearity was addressed by modeling age as a categorical variable.

All statistical analyses were conducted using R version 4.1.1 (R Foundation for Statistical Computing, Vienna, Austria) and RStudio version 1.4.1717.

This study was reported in accordance with the Strengthening the Reporting of Observational Studies in Epidemiology (STROBE) guidelines (12).

### Ethical considerations

The study complies with the principles of the Declaration of Helsinki and received approval from the Comité de Ética en Investigación Biomédica (CEIB), the institutional review board (IRB) of FVL (September 20, 2023/No. 560–2023). As the coordinating institution, the CEIB of FVL waived the requirement for patient consent, given that no interventions were planned for the participants and information was obtained from medical records.

## Results

### General characteristics and comorbidities

**Figure 1:**
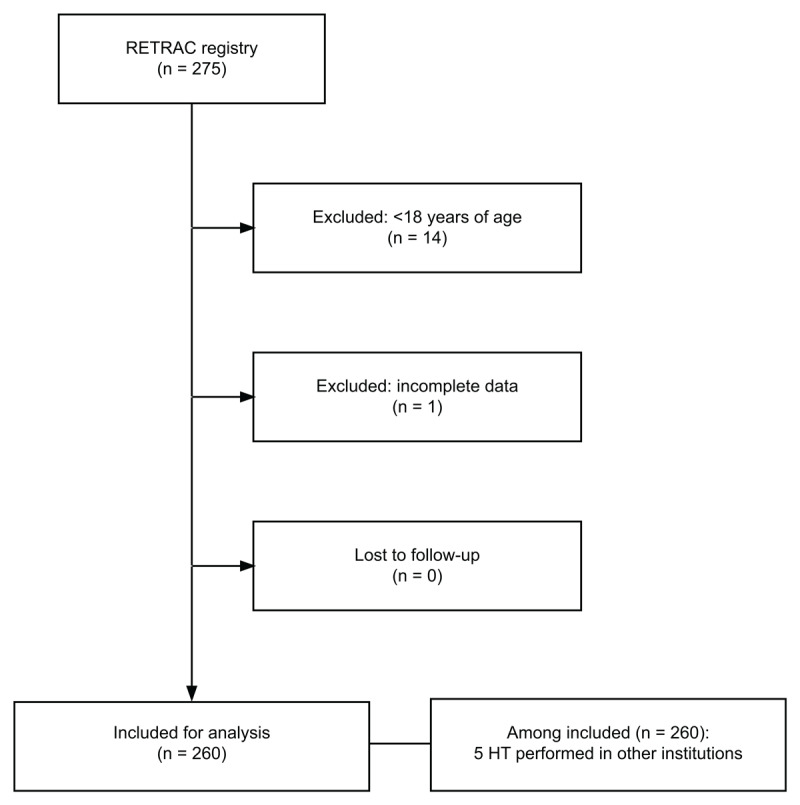
Strobe flow diagram. Abbreviation: HT, heart transplantation.

Among 275 HT patients in the registry, 270 underwent HT at our institution, and we included 260 who met the inclusion criteria (255 transplanted at our institution and 5 with HT performed in other institutions). Furthermore, we excluded 14 patients younger than 18 years of age and 1 patient with incomplete data. We did not exclude patients who were lost to follow-up (Figure 1). The median age was 51 years (interquartile range (IQR): 39.5–59.0), with a predominance of male patients (78%, n = 202 vs 22%, n = 58). The underlying etiologies of HF were idiopathic/dilated cardiomyopathy (IDC) (41.3%, n = 107), IC (27%, n = 70), and valvular heart disease (VHD) (9.7%, n = 25). The most prevalent comorbidities were HTN (48.3%, n = 125), DM (18.9%, n = 49), and CKD (13.1%, n = 34). Other etiologies and comorbidities are described in Table 1.

**Table 1:** Baseline characteristics.


VARIABLE	OVERALL (n = 260)

**Sociodemographic characteristics**	

Age, years	51.0 (39.5, 59.0)

Female	58 (22.3%)

Male	202 (77.7%)

**Main etiology**	

Chagas	7 (2.7%)

Hypertrophic	5 (1.9%)

IDC	107 (41.3%)

IC	70 (27.0%)

Restrictive	5 (1.9%)

VHD	25 (9.7%)

Viral	9 (3.5%)

Others	32 (12.3%)

**Comorbidities**	

HTN	125 (48.3%)

CKD	34 (13.1%)

Chronic obstructive pulmonary disease	6 (2.3%)

Deep venous thrombosis	9 (3.5%)

DM	49 (18.9%)

Neoplasia	7 (2.7%)

Occlusive arterial disease	12 (4.6%)

Pulmonary embolism	20 (7.7%)

Stroke	23 (8.9%)

Transient ischemic attack	9 (3.5%)


Data are shown as n (%) or median (interquartile range). Other categories include arrhythmia, cardiotoxicity, chemotherapy, infiltrative, inherited, and peripartum. Abbreviations: CKD, chronic kidney disease; DM, diabetes mellitus; HTN, hypertension; IC, ischemic cardiomyopathy; IDC, idiopathic/dilated cardiomyopathy; VHD, valvular heart disease.

### Etiologies by age

The <40-year age group exhibited IDC as the predominant etiology (44.8%, n = 30), followed by viral cardiomyopathy (9%, n = 6). In the 40–49-year age group, IDC remained the leading etiology (39.6%, n = 30), while VHD was the second most prevalent (18.9%, n = 10), and IC was the third (17%, n = 9). The 50–59-year age group demonstrated a similar pattern, with IDC (44.7%, n = 38), IC (35.3%, n = 30), and VHD (7.1%, n = 6) as the principal etiologies. Notably, in the oldest patient group (>60 years), IC was the primary etiology (48.1%, n = 26), followed by IDC (33.3%, n = 18) and VHD (7.4%) (Table 2).

**Table 2:** Etiologies by age group.


VARIABLE	<40 (N = 67)	40–49 (N = 53)	50–59 (N = 86)	≥60 (N = 54)

Chagas	2 (3.0%)	3 (5.7%)	1 (1.2%)	1 (1.9%)

Hypertrophic	1 (1.5%)	2 (3.8%)	1 (1.2%)	1 (1.9%)

IDC	30 (44.8%)	21 (39.6%)	38 (44.2%)	18 (33.3%)

IC	5 (7.5%)	9 (17.0%)	30 (34.9%)	26 (48.1%)

Restrictive	1 (1.5%)	3 (5.7%)	1 (1.2%)	0 (0.0%)

VHD	5 (7.5%)	10 (18.9%)	6 (7.0%)	4 (7.4%)

Viral	6 (9.0%)	2 (3.8%)	0 (0.0%)	1 (1.9%)

Others	17 (25.4%)	3 (5.7%)	9 (10.5%)	3 (5.6%)


Data are shown as n (%). Other categories include arrhythmia, cardiotoxicity, chemotherapy, infiltrative, inherited, and peripartum. Abbreviations: IC, ischemic cardiomyopathy; IDC, idiopathic/dilated cardiomyopathy; VHD, valvular heart disease.

### Overall survival and survival time

**Figure 2:**
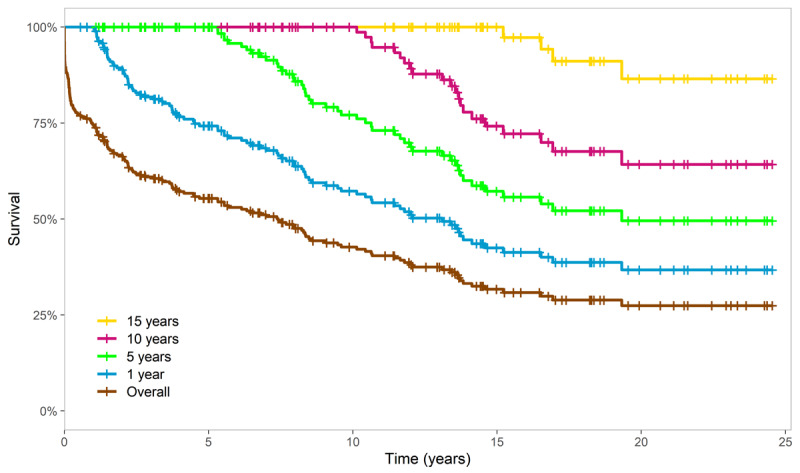
Survival curves based on survival status at different time frames.

After 29 years of patient recruitment, the overall survival was 7.4 years (95% CI: 4.7–9.6). Patients who survived the first year (74.6%) had a median survival of 13 years (95% CI: 8.2–13). Among those who survived 5 years (56.9%), median survival was 19 years. Patients who survived 10 years (46.9%) had a median survival of 26 years. For those who survived 15 years (40.3%), the median survival could not be determined due to the low number of deceased patients. Therefore, the restricted mean survival time was calculated, yielding a value of 25.75 years (Figure 2).

### Survival based on age groups

**Figure 3:**
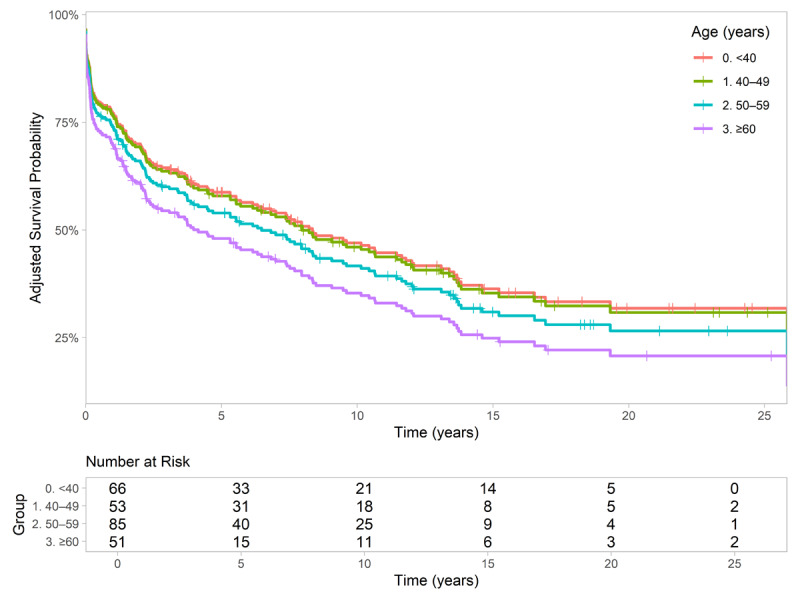
Adjusted age-specific survival curve.

In the age-stratified survival analysis, the 40–49-year age group exhibited the most pronounced reduction in survival, with a median survival of 8.2 years and a mortality rate exceeding 50% at five years of follow-up. Patients aged ≥60 years showed a comparable decline in the survival curve, with a lower median survival of 2.2 years. In contrast, patients younger than 40 years demonstrated the most favorable survival, with a median survival of 8.4 years. The 50–59-year age group had an intermediate survival profile, with a median survival of 6.5 years. These differences were statistically significant (p = 0.038) (Supplementary 4). However, after stratifying the survival curves by age group and adjusting for DM and CKD using direct standardization, no significant differences were observed among the age groups. Notably, the previously elevated mortality rate in the 40–49-year age group was no longer apparent (Figure 3).

### Survival based on sex

In the sex-related survival curve analysis, we observed statistical significance only in the <40-year age group (p = 0.0078). Within this group, females exhibited a five-year mortality rate of approximately 75%, whereas males demonstrated a more favorable prognosis, with a five-year mortality rate of 25% (Supplementary 5). Similarly, a specific 10-year survival analysis of the ≥60-year age group revealed a comparable pattern, with males having a higher survival rate, while females exhibited a more rapid decline in survival (p = 0.034) (Supplementary 6). The survival curves for the 40–49-year and 50–59-year age groups did not reach statistical significance (Supplementary 5).

### Survival based on HT time periods

In the analysis of HT time periods, survival curves were generated for six five-year intervals from 1995 to 2020. The largest sample groups were from 2006 to 2010 and 2016 to 2020, comprising 24.2% and 20.7% of the total population, respectively. Detailed information on group sample sizes and patients’ current status is provided in Supplementary 7.

**Figure 4:**
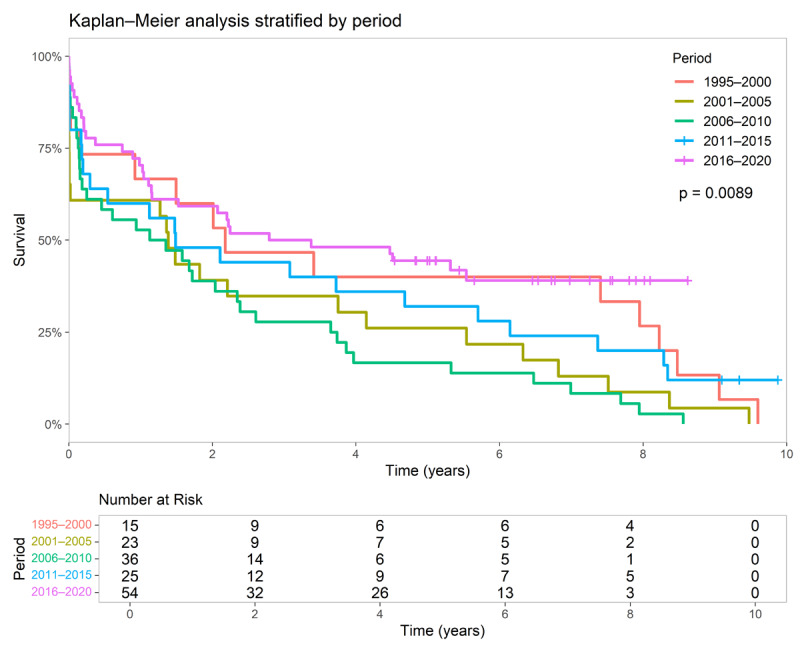
Survival curves stratified by five-year periods based on the timing of the HT. *Patients who underwent HT after 2020 were excluded from this analysis because they had not completed five years of follow-up at the time of inclusion. Abbreviation: HT, heart transplantation.

The 2016–2020 group demonstrated the highest survival rates, with 50% survival at four years and approximately 40% at eight years of follow-up, despite being in the early stages of the COVID-19 pandemic. Similarly, the 1995–2000 group exhibited comparable survival rates at the same follow-up time points. In contrast, the 2006–2010 group had the lowest survival, with less than 25% at four years. The remaining time periods had five-year survival rates exceeding 25% (Figure 4). This analysis was statistically significant (p = 0.0089). Detailed age data of five-year periods are provided in Supplementary 8.

### Perioperative and donor risk factors for post-transplant mortality

**Figure 5:**
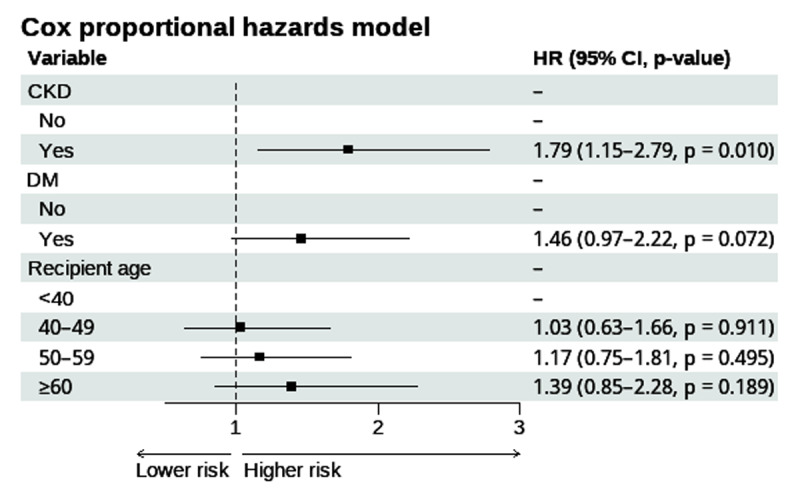
Forest plot parsimonious model. *Events-per-variable ratio of 53. Abbreviations: CKD, chronic kidney disease; DM, diabetes mellitus; HR, hazard ratio.

By the Cox multivariate model, the only significant risk factor identified was CKD, exhibiting a 79% higher risk of mortality compared to those without a previous CKD (hazard ratio (HR) = 1.79; IC: 1.15–2.79; p = 0.010) (Figure 5). Other risk factors, including age group, history of other comorbidities, etiology, prolonged ischemic time (>150–200 minutes), diminished post-transplant left ventricular ejection fraction (<50%), and support management, did not yield statistically significant results (Tables 3 and 4). A saturated model is provided in Supplementary 9.

**Table 3:** Donor and perioperative risk factors for post-transplant mortality.


VARIABLE	HR UNADJUSTED (n = 260)	CI (95%) UNADJUSTED	p-VALUE UNADJUSTED	HR MODEL 2 (n = 255)	CI (95%) MODEL 2	p-VALUE MODEL 2

**Perioperative variable**						

Inotropics	1.32	(0.96–1.83)	0.090	–	–	–

**Ischemic time, minutes**						

≤200	–	–	–	–	–	–

201–400	1.1	(0.84–1.62)	0.347	–	–	–

>400	1.31	(0.48–3.57)	0.601	–	–	–

**Ischemic time, minutes**						

≤150	–	–	–	–	–	–

>150	1.27	(0.89–1.82)	0.188	–	–	–

**Year of transplant**						

1995–2000	–	–	–	–	–	–

2001–2005	1.14	(0.65–2.00)	0.649	–	–	–

2006–2010	1.12	(0.67–1.87)	0.673	–	–	–

2011–2015	0.99	(0.55–1.76)	0.968	–	–	–

2016–2020	1.52	(0.87–2.66)	0.145	–	–	–

2021–2024	1.02	(0.48–2.21)	0.950	–	–	–

**Donor variables**						

Donor age of death	1.00	(0.98–1.02)	0.880	–	–	–

Donor DM	0.76	(0.11–5.49)	0.789	–	–	–

Donor HTN	0.95	(0.24–3.85)	0.944	–	–	–

Donor smoking	0.56	(0.29–1.07)	0.080	–	–	–


*Both Tables 3 and 4 are based on the same parsimonious model (Model 2). Abbreviations: DM, diabetes mellitus; HR, hazard ratio; HTN, hypertension.

**Table 4:** Recipient risk factors for post-transplant mortality.


VARIABLE	HR UNADJUSTED (n = 260)	CI (95%) UNADJUSTED	p-VALUE UNADJUSTED	HR MODEL 2 (n = 255)	CI (95%) MODEL 2	p-VALUE MODEL 2

**Recipient variables**						

CKD	2.01	(1.30–3.10)	0.002	1.79	(1.15–2.79)	0.010

Creatinine	1.05	(0.91–1.22)	0.511	–	–	–

DM	1.69	(1.15–2.49)	0.008	1.46	(0.97–2.22)	0.072

HTN	0.95	(0.70–1.30)	0.767	–	–	–

**LVEF categorized**						

Reduced (<40%)	–	–	–	–	–	–

Mildly reduced (40%–49%)	0.78	(0.19–3.16)	0.731	–	–	–

Preserved (≥50%)	1.33	(0.54–3.25)	0.535	–	–	–

**Recipient age, years**						

<40	–	–	–	–	–	–

40–49	1.02	(0.63–1.64)	0.936	1.03	(0.63–1.66)	0.911

50–59	1.32	(0.87–2.00)	0.195	1.17	(0.75–1.81)	0.495

≥60	1.63	(1.03–2.58)	0.350	1.39	(0.85–2.28)	0.189

**Sex**						

Female	–	–	–	–	–	–

Male	0.75	(0.53–1.07)	0.113	–	–	–

**Recipient previous heart disease**						

IC	–	–	–	–	–	–

IDH	0.80	(0.55–1.16)	0.239	–	–	–

Other underlying heart disease	0.71	(0.45–1.13)	0.152	–	–	–

VHC	1.08	(0.62–1.87)	0.794	–	–	–


*Both Tables 3 and 4 are based on the same parsimonious model (Model 2). Abbreviations: CKD, chronic kidney disease; DM, diabetes mellitus; HR, hazard ratio; HTN, hypertension; IC, ischemic cardiomyopathy; IDC, idiopathic/dilated cardiomyopathy; LVEF, left ventricular ejection fraction; VHD, valvular heart disease.

Furthermore, the global and individual Schoenfeld tests indicated that the model remains valid over time, supporting proportional hazards assumptions (Supplementary 10 and 11). Additionally, an assessment of differences in the β coefficients (DFBETAs) revealed no materially influential observations (Supplementary 12).

## Discussion

This cohort study provides evidence of the transplant center’s experience in a southwestern healthcare facility in Colombia. The most prevalent comorbidities observed in HT patients were HTN, DM, and CKD. The primary etiologies were IDC and IC. In the survival analysis, we identified lower survival rates in the 40–49-year age group; however, this effect was attenuated after adjustment. Furthermore, male individuals had a better prognosis among young patients. Similarly, the most recent patient cohort (2016–2020) demonstrated the highest five-year survival rates, contrasting with earlier periods. Moreover, CKD emerged as the primary mortality risk factor in our population, highlighting its significance in patient care. These study findings enhance understanding of the HT population and their specific characteristics, contributing to improved risk optimization and long-term management strategies.

### Sociodemographic characterization

The sociodemographic characteristics of HT patients are generally similar worldwide, despite variations across continents and economic strata. In our study, the median age of HT patients was 51.0 years, with a higher proportion of males (77.7%) compared to females. This aligns with ISHLT data, where the majority of patients were from North America and Europe, with a median age of 54.0 years and a predominance of males (77.9%) (5). Suarez-Pierre et al. reported similar findings in North America, where 29,000 HT patients had a median age of 52.7 years and a male population of 75% (13). In contrast, Lee et al. reported lower median recipient ages in Taiwan, Hong Kong, Singapore, Korea, and Japan while maintaining the same pattern in sex distribution (14). A single-center Brazilian study observed a median age of 46.6 years, with 74.9% of patients being male (15). In Colombia, data from Medellín showed a similar median age of 49 years, with 84.1% of patients being male (8). These findings indicate that the demographics of HT patients are largely consistent worldwide, with our population trends fully concordant with those in North America, Europe, and other Colombian studies, and partially concordant with Asian cohorts. Furthermore, these results highlight potential sex disparities, with a clear predominance of HT in the male population (16,17).

### Etiology of HF

The main etiologies of HF are consistent with the global distribution, with a notable prevalence of IDC, IC, and VHD. In our study, the most prevalent etiologies were IDC, IC, and VHD. Similarly, the ISHLT registry reports a distribution of IDC (48.1%), IC (40.61%), and VHD (3.45%) (5). In the same way, in Brazil, reports indicate IDC (33%), Chagasic cardiomyopathy (18%), and IC (14.3%), highlighting the notably high prevalence of Chagas disease (15). Likewise, an HT study conducted in Medellín identified IC (36.1%), IDC (31.2%), and VHD (9.2%) as the predominant causes (8). These findings indicate a consistent pattern across different populations, highlighting the concordance between our research and previous studies. Notably, in our study, the predominance of IC in patients older than 60 years aligns with trends observed in the aforementioned studies, suggesting that this age group is particularly affected by IC.

### Comorbidities

HTN is the most prevalent comorbidity across different HT patient populations; however, other comorbidities may vary across registries. In our patients, the prevalence of HTN was 48.3%, followed by DM (18.9%) and CKD (13.1%). According to Lund et al.’s analysis of the ISHLT registry, the principal comorbidities were HTN (51.2%) and DM (26.3%) (18). Similarly, Vinck et al. identified HTN as the most common comorbidity, consistent with our study and the ISHLT registry. Additionally, Vinck et al. reported lower prevalence rates of DM and CKD compared to our study (8). These results underscore both the similarities and distinctions in the clinical characteristics of our population compared to global data and highlight the significance of HTN within the cardiovascular disease spectrum.

### Survival analysis

Despite similar age distribution, main etiologies, and a partially similar pattern of comorbidities in HT patients worldwide, survival rates and outcomes vary across populations. According to the ISHLT registry, the overall median survival for HT patients was 11.3 years. Age-specific analysis revealed that younger patients had higher survival rates, with those under 40 years having a median survival of 13.2 years, in contrast to patients older than 60 years, who had the lowest median survival of 9.7 years. Sex-based comparisons showed differences displaying higher survival length for females; however, the clinical variations were minimal (male: 11.1 years vs female: 11.8 years). Additionally, analysis of HT periods demonstrated improvements in survival rates over time, with patients transplanted between 1992 and 2000 having a median survival of 10.2 years, compared to 12.1 years for those transplanted between 2001 and 2009 (5). Suarez-Pierre et al. reported overall survival rates at 1, 5, and 10 years of 90%, 77%, and 59%, respectively. In age-specific analysis, patients under 40 years exhibited the highest conditional survival at 5 years post-transplant, whereas older patients (>60 years) had the lowest survival rates. Interestingly, sex analysis revealed that among younger patients, males had better conditional five-year survival rates than females, whereas among older patients, females had better survival rates than males (13). In the study by Ferraz et al., survival rates at 1, 5, and 10 years were 68.1%, 58.0%, and 40.8%, respectively; moreover, improved survival was observed in more recent HT cohorts (15). Researchers in Medellín, Colombia, reported survival rates at 1, 5, 10, and 15 years of 71.3%, 50.7%, 36.5%, and 24.2%, respectively. Their study included a sex-based comparison, which demonstrated similar survival rates for both sexes. Furthermore, in the HT five-year transplant-period analysis—where the study classified patients into eight five-year groups from 1985 to 2020—higher survival times were observed in patients who underwent transplantation in more recent years. This finding is likely related to advancements in post-transplant clinical management, including improvements in immunosuppressive therapy and HF supportive care (8).

Our population’s survival is lower than that of ISHLT patients, with a median difference of 3.9 years (5). In our cohort, survival rates at 1, 5, and 10 years were 74.6%, 56.9%, and 46.9%, respectively. Similarly, our survival rates are lower compared to North American patients in the Suarez-Pierre study, with differences at 1, 5, and 10 years of 15.4%, 20.1%, and 12.1%, respectively, maintaining a similar disparity at the 10-year follow-up (13). Furthermore, our age-specific survival exhibited a partially similar pattern, with worse outcomes in older patients. Patients under 40 years had the highest median survival, while those over 60 had the lowest. However, our analysis revealed an unusual survival trend in the 40–49-year age group, where these patients experienced the most rapid decline in survival rates, with more than 50% mortality at 5 years post-HT. In the adjusted Kaplan–Meier analysis, this anomaly attenuated, aligning with prior studies. Therefore, this effect could be a product of our small sample and a limited statistical power.

When comparing HT patients from single-center studies in Brazil and Colombia, we observed that our population exhibited higher survival percentages compared to other studies within our own country but lower survival rates relative to Brazil. The survival differences at 1, 5, and 10 years, when contrasted with the Brazilian study, were 15.4%, 20.1%, and 12.1%, respectively, with higher survival among Brazilian patients (15). In the comparison between Cali and Medellín, our population demonstrated higher survival percentages. The survival differences at 1, 5, 10, and 15 years between Cali and Medellín were 3.3%, 6.2%, 10.4%, and 16.1%, respectively, indicating a significant disparity that widened over time (8). These findings emphasize the heterogeneity between HT institutions located in different regions of the same LATAM country and underscore the necessity for more comprehensive studies to further characterize these distinct populations.

Survival rates varied according to sex distribution across different age groups. Younger male patients (<40 years) exhibited higher survival rates than their female counterparts. Similarly, a 10-year survival analysis of older patients demonstrated a comparable sex-related survival pattern. However, it is important to acknowledge potential population disparities, as male patients constituted the predominant demographic in our study, which may have influenced the results. In contrast, Suarez-Pierre et al. observed a similar survival trend in younger patients; however, their study reported better survival rates among older female patients (13). It is crucial to consider the methodological differences between the two studies. While Suarez-Pierre et al. employed a five-year conditional survival analysis, we conducted a non-conditional survival analysis throughout our inclusion period, making direct comparisons challenging.

In the analysis of the transplant date five-year interval, our findings revealed a partially distinct pattern compared to the results of previously mentioned studies (5,8). Notably, our research did not demonstrate higher survival rates in all of the most recent HT transplant patients. We observed that the newest cohort (2016–2020) had the highest survival rates, which aligns with findings from the ISHLT registry, the study by Vinck et al., and Ferraz et al. However, the earlier cohort (1995–2000) exhibited the second-highest survival rate, which contradicts the trends reported in these studies (5,8,15). This distinct pattern in the earliest cohort can be attributed to historical differences in HT criteria, as younger patients were prioritized for transplantation, and patients over 60 years of age were considered contraindicated for the procedure (19,20,21). In addition, donor and recipient age are well-established determinants of post-HT outcomes; thus, differences in age distributions across eras may have contributed to these findings (22,23,24).

### Mortality risk factors

A history of CKD is a significant mortality risk factor among HT patients and is associated with poor prognosis. In our study, patients with a history of CKD had a 79% higher risk of mortality compared to those without this comorbidity. Several researchers have identified a history of CKD as a risk factor for post-HT malignancies, including solid-organ malignancies (25,26). Similarly, CKD has been established as a known risk factor for mortality, advanced CKD post-HT, and subsequent kidney transplantation (27,28,29). Therefore, our results align with the existing literature, highlighting a pattern in the implications of CKD pre-HT as a critical factor for improving long-term survival and outcomes in this population. From a practical perspective, our findings emphasize the need for systematic renal risk stratification before HT and structured post-HT surveillance to mitigate downstream complications.

### Strengths and limitations

The strengths of this study include its cohort design and an extensive timeline encompassing 29 years of data collection. Additionally, this study addresses a significant information gap regarding HT patients in the southwestern region of Colombia, establishing it as one of the most valuable regional data sources. We conducted comprehensive statistical analyses, ensuring our findings are easily comparable to both national and international datasets. The limitations of this study include the use of retrospective data that may introduce information bias. Furthermore, while this research offers valuable regional insights, the single-institution design at a national level hinders the generalization of findings to both national and international populations. Moreover, our sample may not have been sufficient to detect small differences among HT patients. Finally, we assessed all-cause mortality as the event of interest; thus, our study did not differentiate cardiovascular causes of death from other etiologies.

## Conclusions and Future Perspectives

Patients with HT in Colombia exhibit characteristics, comorbidities, and underlying cardiac conditions similar to those reported in both national and international registries. However, clinical outcomes and survival rates remain distinct, underscoring the heterogeneous nature of different populations. These findings highlight the need for region-specific and comprehensive data to develop tailored management and follow-up strategies aimed at improving survival and clinical outcomes. Additionally, understanding the risk factors in our population, such as CKD, is crucial for the prevention and targeted management of conditions that affect our patients. This further supports the need to develop multi-center regional registries capable of providing comprehensive and comparable data to guide evidence-based, context-specific strategies for improving HT outcomes in LATAM.

## Use of Artificial Intelligence

Artificial intelligence programs (large language models) were used for proofreading and spell check.

## Data Accessibility Statement

The data presented in this study are included in the article and its Supplementary Material. De-identified participant-level data underlying the reported results, the data dictionary, and the analytic code can be made available by the corresponding author upon reasonable request, subject to institutional approval and execution of a data use agreement, in accordance with applicable ethical and privacy regulations.

## Additional File

The additional file for this article can be found as follows:

10.5334/gh.1520.s1 Supplementary Material. Supplementary 1 to 12.
