# Supplementary material for: Survival in Adult Patients Undergoing Heart Transplantation 1995–2024: A Report of the RETRAC Registry
Source: Glob Heart. 2026 Feb 3;21(1):7. doi: 10.5334/gh.1520 (PMC12880014; doi:10.5334/gh.1520)

SUPPLEMENTARY

Supplementary 1: Events per variable

Variable	Alive (n= 100)	Deceased (n= 160)
**Recipient variables**		
LVEF categorized		
Preserved (>=50%)	2 (2.0%)	5 (3.3%)
Mildly reduced (40%-49%)	2 (2.0%)	2 (1.3%)
Reduced (<40%)	95 (96%)	146 (95.4%)
Inotropics	63 (64.3%)	96 (60.8%)
Ischemic time		
<=200 minutes	52 (53.1%)	88 (56.4%)
201-400 minutes	44 (44.9%)	64 (41.0%)
>400 minutes	2 (2.0%)	4 (2.6%)
Ischemic time 2		
<=150 minutes	30 (30.6%)	41 (26.3%)
>150 minutes	68 (69.4%)	115 (73.7%)

Variable	Alive (n= 100)	Deceased (n= 160)
Recipient Age		
<40	30 (30%)	37 (23%)
40-49	22 (22%)	31 (19.4%)
50-59	31 (31%)	55 (34.4%)
≥60	17 (17%)	37 (23.1%)
Recipient CKD	9 (9.0%)	25 (16.1%)
Recipient DM	16 (16.0%)	33 (20.8%)
Recipient sex		
Female	16 (16%)	42 (26.3%)
Male	84 (84.0%)	118 (73.8%)
Recipient HTN	50 (50.0%)	75 (47.2%)
Recipient previous heart disease		
Idiopathic dilated cardiomyopathy	41 (41.0%)	66 (41.3%)
Ischemic heart disease	21 (21.0%)	49 (30.6%)

Variable	Alive (n= 100)	Deceased (n= 160)
Other underlying heart disease	30 (30%)	28 (17.5%)
Valvulopathy	8 (8.0%)	17 (10.6%)
Year of transplant		
1995-2000	7 (7.0%)	23 (14.4%)
2001-2005	11 (11.0%)	27 (16.9%)
2006-2010	20 (20.0%)	43 (26.9%)
2011-2015	19 (19.0%)	25 (15.6%)
2016-2020	22 (22.0%)	32 (20.0%)
2021-2024	21 (21%)	10 (6.3%)
Donor variables		
Donor DM	1 (1.5%)	1 (0.8%)
Donor HTN	1 (1.5%)	1 (1.5%)
Donor smoking	10 (14.7%)	10 (7.5%)

Abbreviations: CKD: Chronic kidney disease, DM: Diabetes mellitus, HR: Hazard ratio, HTN: Hypertension, LVEF: Left ventricular ejection fraction

Supplementary 2: Missing data

Variable	n	n missing	% missing
Age, years	260	0	0,0
Age group	260	0	0,0
Sex	260	0	0,0
Main etiology	260	0	0,0
HTN	259	1	0,4
CKD	255	5	1,9
Chronic obstructive pulmonary disease	259	1	0,4
Deep venous thrombosis/Pulmonary embolism	259	1	0,4
DM	259	1	0,4
Neoplasm	259	1	0,4
Occlusive arterial disease	259	1	0,4
Stroke/Transient ischemic attack	259	1	0,4
LVEF categorized	252	8	3,1
Creatinine	254	6	2,3
Inotropics	256	4	1,5
Ischemic time 2	254	6	2,3

Year of transplant	260	0	0,0
Donor age of death	201	59	22,7
Donor DM	201	59	22,7
Donor HTN	201	59	22,7
Donor smoking	201	59	22,7

Abbreviations: CKD: Chronic kidney disease, DM: Diabetes mellitus, HR: Hazard ratio, HTN: Hypertension, LVEF: Left ventricular ejection fraction

Supplementary 3: GVIF for the Parsimonious Multivariable Cox Proportional Hazards Model

Variable	GVIF	DF	GVIF (1/(2×df))
Age groups	1.16	3	1.02
DM	1.14	1	1.07
CKD	1.03	1	1.02

Abbreviations: CKD: Chronic kidney disease, DF: degrees of freedom, DM: Diabetes mellitus, GVIF: Generalized Variance Inflation Factor.

*A GVIF value close to 1 indicates no multicollinearity. Values below 2 indicate that multicollinearity is not affecting the stability of the model coefficients

Supplementary 4: Unadjusted Age-specific survival curve

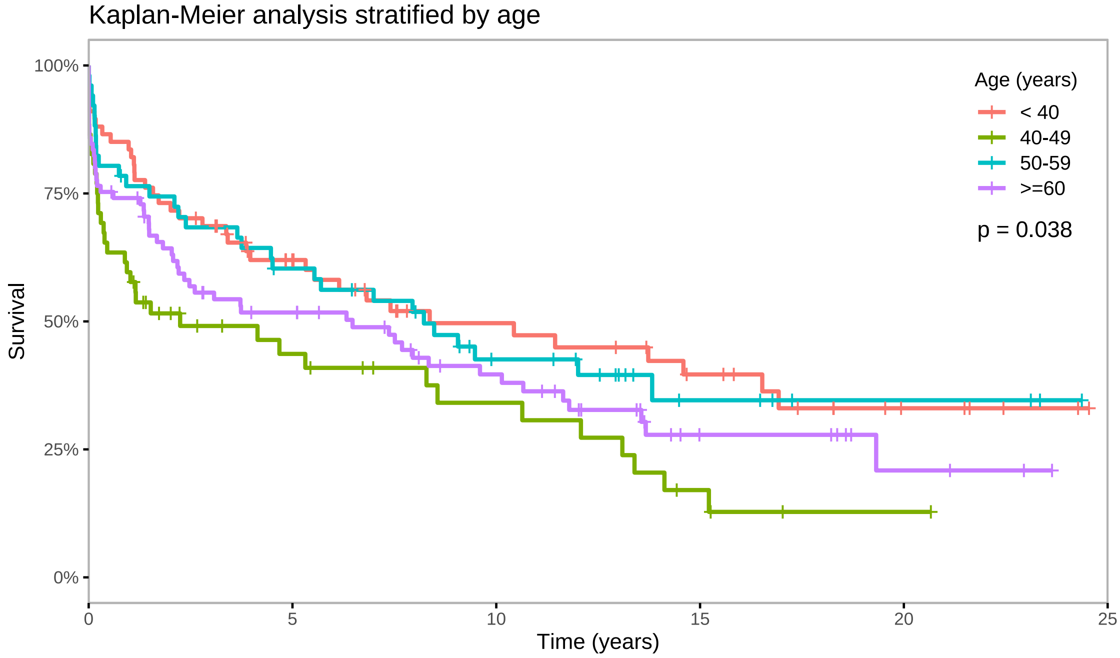

Supplementary 5: Sex-related survival curves

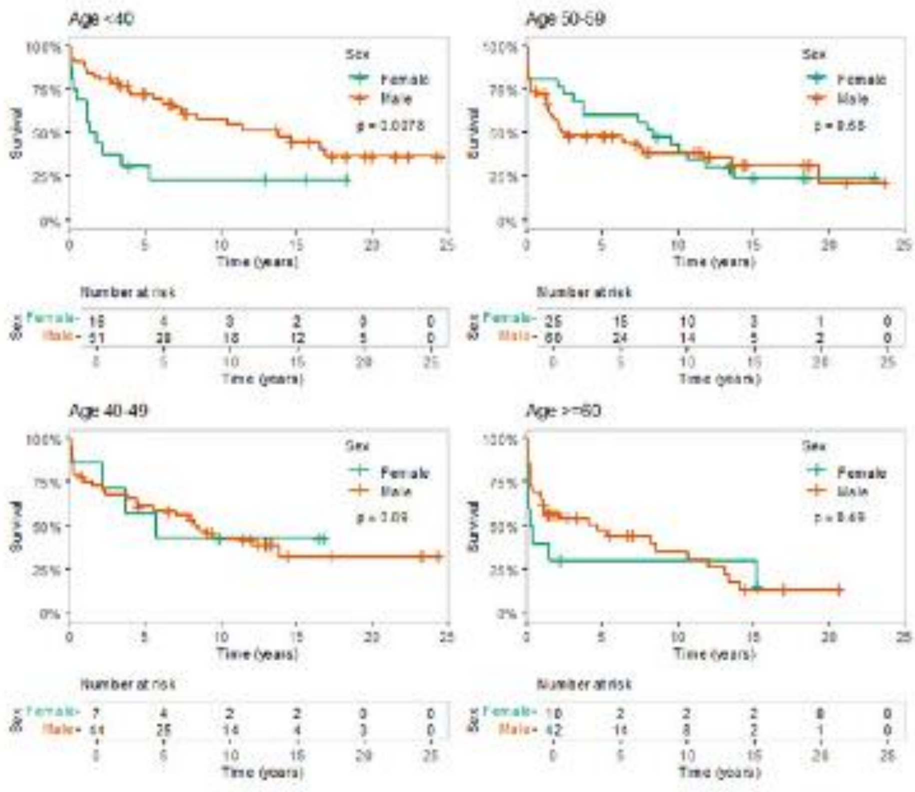

Supplementary 6: a) Overall sex related survival curve and b) Sex-related survival curve for the ≥60 years group: Cut-off 10 years

a)

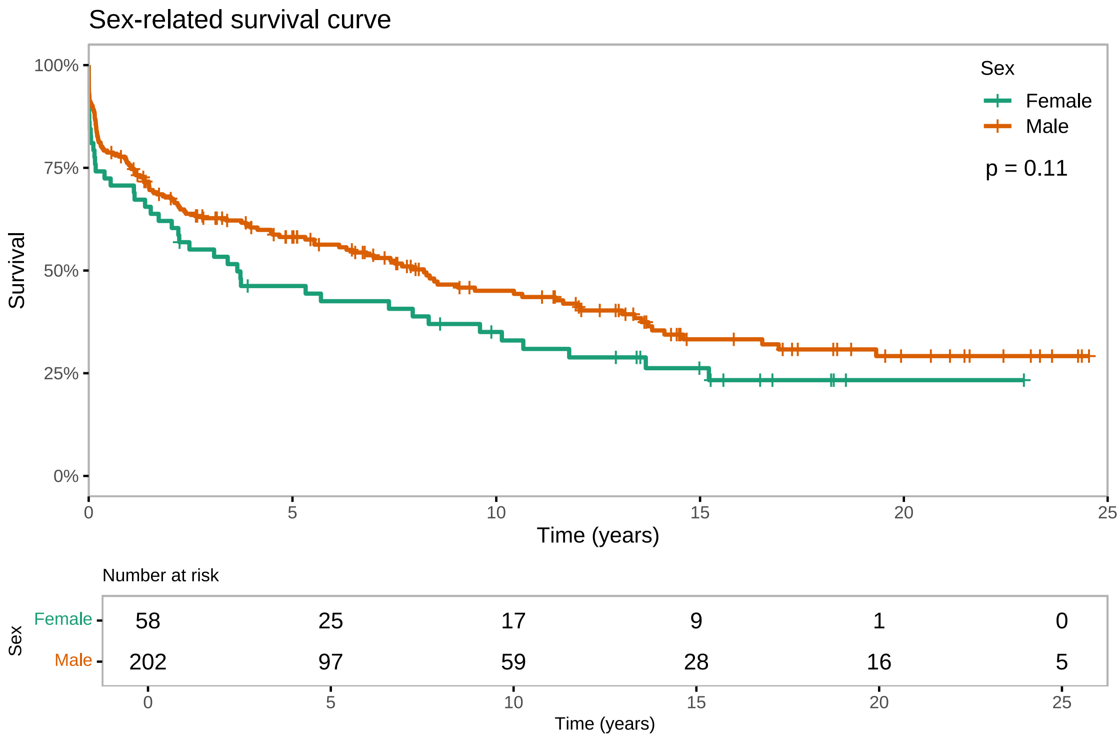

b)

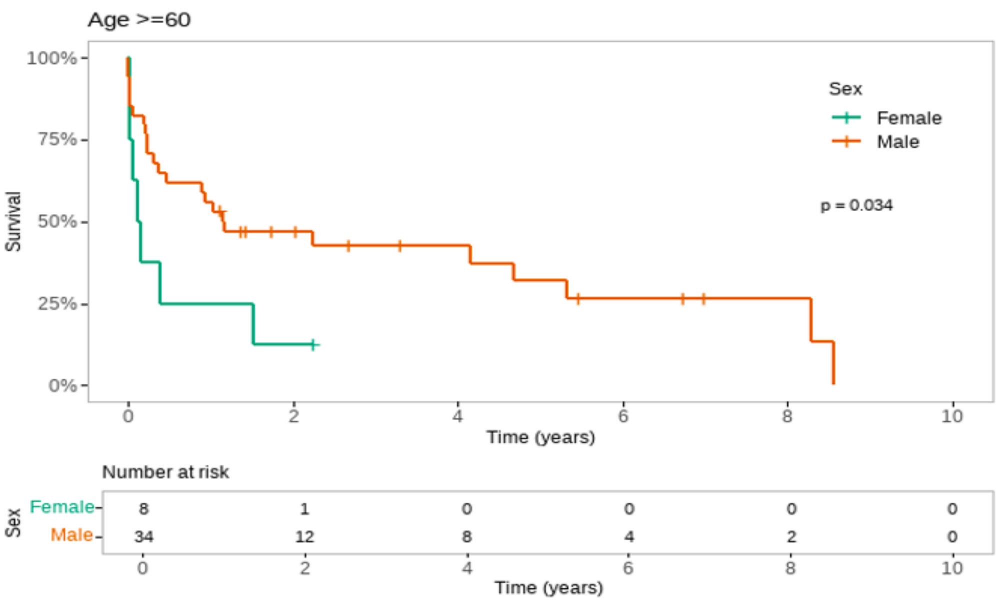

Supplementary 7: Current Status of Heart Transplant Patients Based on 5-Year Transplant Periods

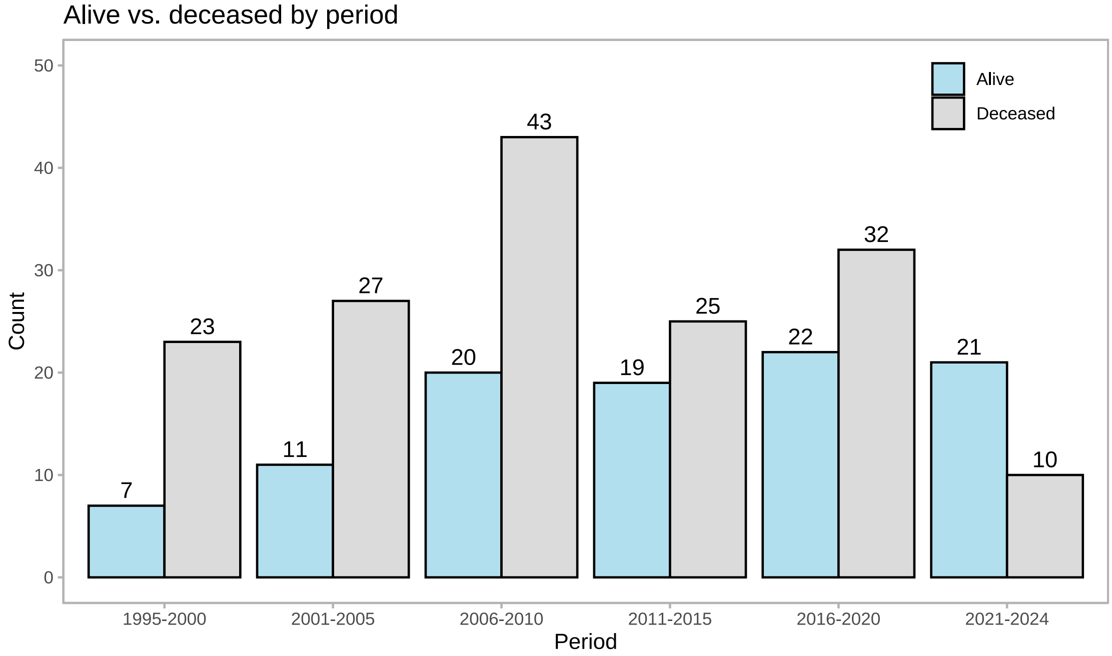

Supplementary 8: Age data of 5-year periods based on the timing of the heart transplant.

Variable	1995-2000<br>N = 30[1]	2001-2005<br>N = 38[1]	2006-2010<br>N = 63[1]	2011-2015<br>N = 44[1]	2016-2020<br>N = 54[1]	2021-2024<br>N = 31[1]
Age	48.9 (39.4, 58.2)	49.4 (34.2, 58.1)	51.8 (40.5, 58.5)	51.6 (44.8, 56.4)	49.7 (35.5, 61.2)	58.5 (40.0, 62.5)

[1] Median (Q1, Q3)

Supplementary 9: Mortality risk factors - Model 1

Variable	HR Unadjusted	CI (95%) Unadjusted	p-value Unadjusted	HR Model 1 (n=247)	CI (95%) Model 1	p-value Model 1
Recipient variables						
Creatinine	1.05	(0.91-1.22)	0.511	-	-	-
LVEF categorized						
Preserved (>=50%)	1.33	(0.54-3.25)	0.535	-	-	-
Mildly reduced (40%-49%)	0.78	(0.19-3.16)	0.731	-	-	-
Reduced (<40%)	-	-	-	-	-	-
Inotropics	1.32	(0.96-1.83)	0.090	1.21	(0.86-1.72)	0.275
Ischemic time						
<=200 minutes	-	-	-	-	-	-
201-400 minutes	1.1	(0.84-1.62)	0.347	-	-	-
>400 minutes	1.31	(0.48-3.57)	0.601	-	-	-

Variable	HR Unadjusted	CI (95%) Unadjusted	p-value Unadjusted	HR Model 1 (n=247)	CI (95%) Model 1	p-value Model 1
Ischemic time 2						
<=150 minutes	-	-	-	-	-	-
>150 minutes	1.27	(0.89-1.82)	0.188	1.10	(0.76-1.60)	0.611
Recipient Age						
<40	-	-	-	-	-	-
40-49	1.02	(0.63-1.64)	0.936	1.01	(0.61-1.66)	0.982
50-59	1.32	(0.87-2.00)	0.195	1.19	(0.73-1.92)	0.485
≥60	1.63	(1.03-2.58)	0.350	1.36	(0.80-2.31)	0.249
Recipient CKD	2.01	(1.30-3.10)	0.002	1.64	(1.04-2.57)	0.033

Variable	HR Unadjusted	CI (95%) Unadjusted	p-value Unadjusted	HR Model 1 (n=247)	CI (95%) Model 1	p-value Model 1
Recipient DM	1.69	(1.15-2.49)	0.008	1.46	(0.95-2.24)	0.085
Recipient sex						
Female	-	-	-	-	-	-
Male	0.75	(0.53-1.07)	0.113	0.77	(0.52-1.15)	0.203
Recipient HTN	0.95	(0.70-1.30)	0.767	-	-	-
Recipient previous heart disease						
Idiopathic dilated cardiomyopathy	0.80	(0.55-1.16)	0.239	0.93	(0.62-1.39)	0.719
Ischemic heart disease	-	-	-	-	-	-
Other underlying heart disease	0.71	(0.45-1.13)	0.152	0.82	(0.48-1.40)	0.469

Variable	HR Unadjusted	CI (95%) Unadjusted	p-value Unadjusted	HR Model 1 (n=247)	CI (95%) Model 1	p-value Model 1
Valvulopathy	1.08	(0.62-1.87)	0.794	1.19	(0.62-2.27)	0.598
Year of transplant						
1995-2000	-	-	-	-	-	-
2001-2005	1.14	(0.65-2.00)	0.649	-	-	-
2006-2010	1.12	(0.67-1.87)	0.673	-	-	-
2011-2015	0.99	(0.55-1.76)	0.968	-	-	-
2016-2020	1.52	(0.87-2.66)	0.145	-	-	-
2021-2024	1.02	(0.48-2.21)	0.950	-	-	-
Donor variables						
Donor age of death	1.00	(0.98-1.02)	0.880	-	-	-
Donor DM	0.76	(0.11-5.49)	0.789	-	-	-
Donor HTN	0.95	(0.24-3.85)	0.944	-	-	-

Variable	HR Unadjusted	CI (95%) Unadjusted	p-value Unadjusted	HR Model 1 (n=247)	CI (95%) Model 1	p-value Model 1
Donor smoking	0.56	(0.29-1.07)	0.080	-	-	-

Abbreviations: CKD: Chronic kidney disease, DM: Diabetes mellitus, HR: Hazard ratio, HTN: Hypertension, LVEF: Left ventricular ejection fraction

Supplementary 10: Schoenfeld test saturated model

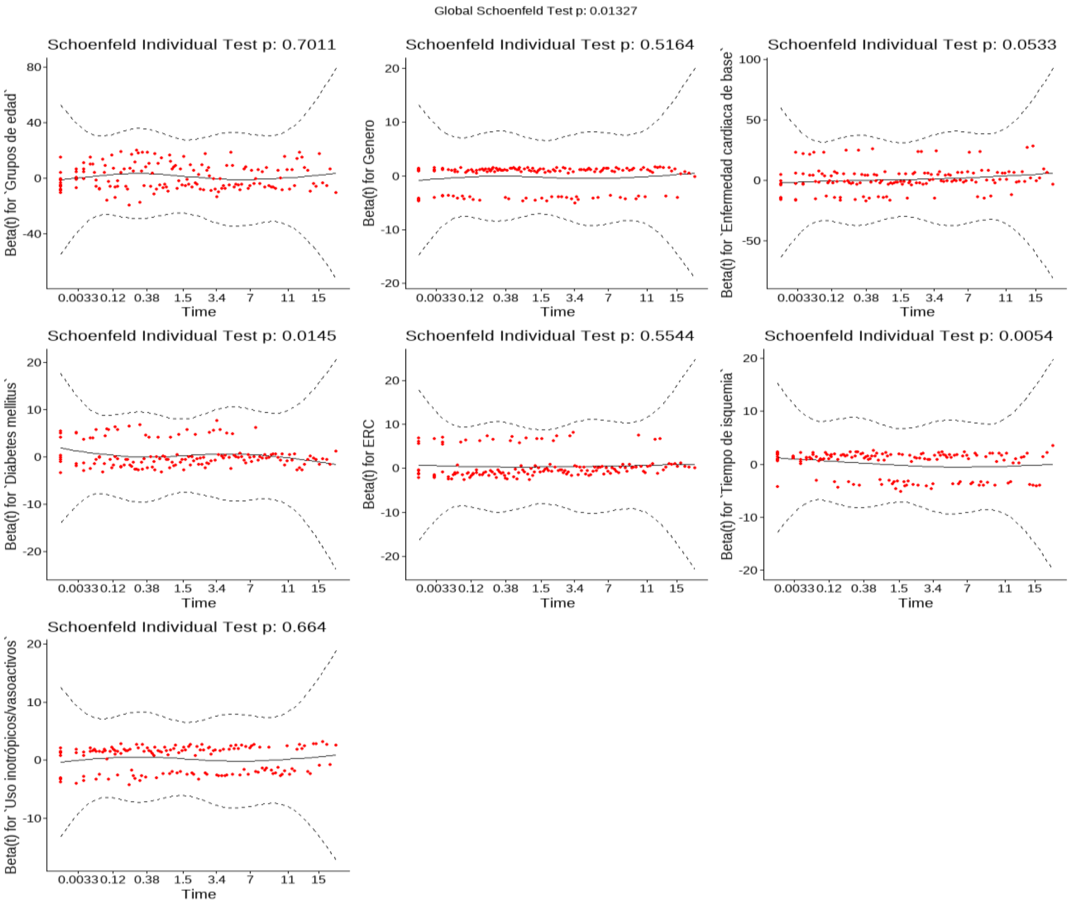

Supplementary 11: Schoenfeld test parsimonious model

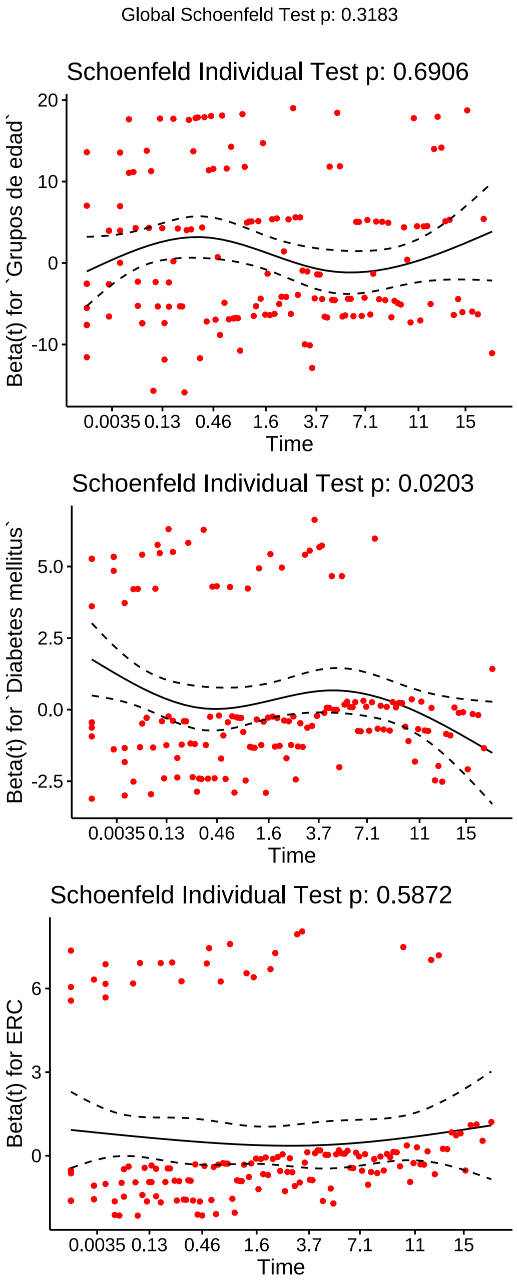

Supplementary 12: Difference in BETA coefficient (DFBETA) Residual Plots for Cox Proportional Hazards Model

a)

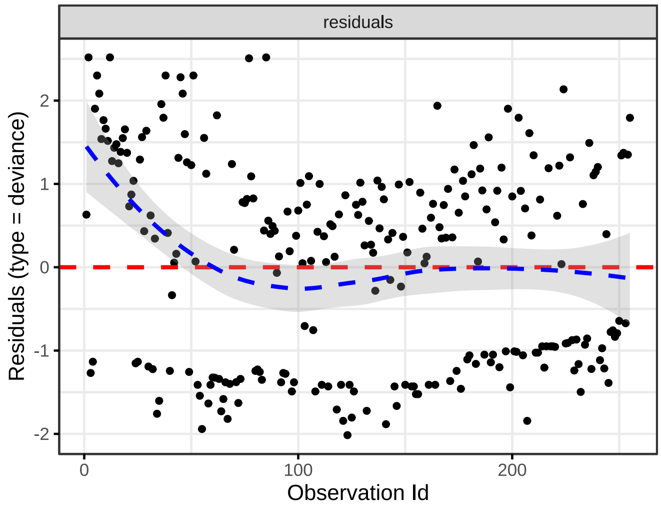

b)

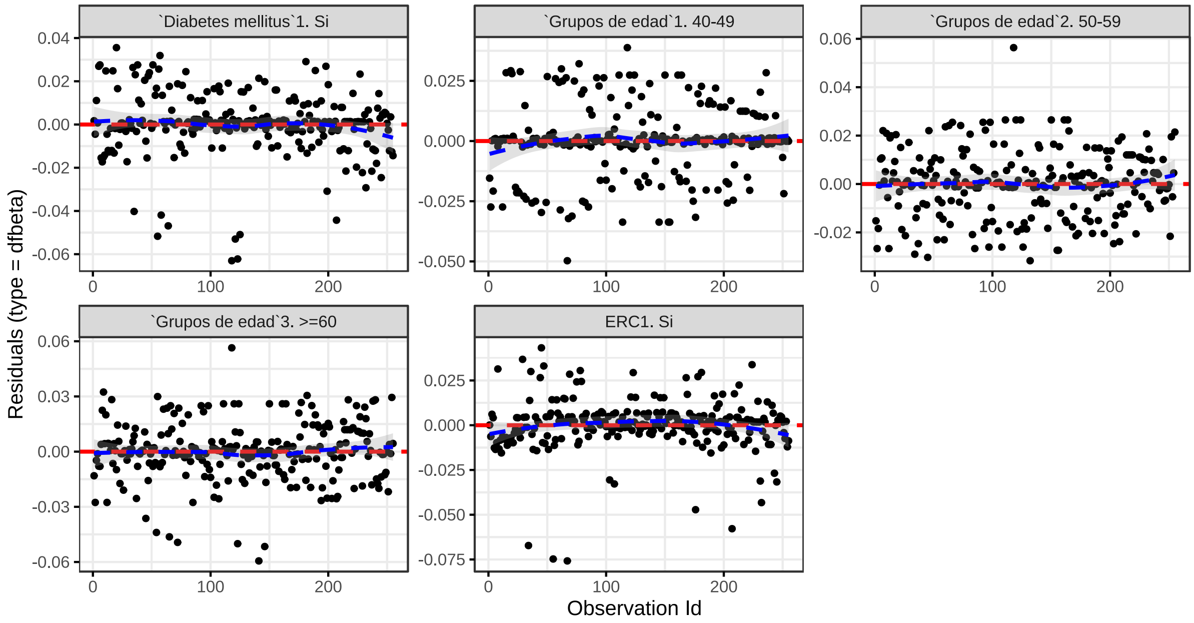

Supplement: Supplementary Material. — Supplementary 1 to 12. [file gh-21-1-1520-s1.pdf]
